# Potential association of cluster headache with certain COVID‐19 vaccines: An assessment from the pharmacovigilance databases

**DOI:** 10.1111/head.70170

**Published:** 2026-07-06

**Authors:** Elise K. Van Obberghen, Laura Parigny, Nouha Ben‐Othman, Elliot Ewig, Milou‐Daniel Drici, Alexandre O. Gérard, Sophie Gautier, Michel Lanteri‐Minet

**Affiliations:** ^1^ Pain Department UR2CA and FHU InovPain, CHU Nice and Université Côte d'Azur Nice France; ^2^ Pharmacology Department, Pharmacovigilance and Addictovigilance Center, French Addictovigilance Network CHU de Lille Lille France; ^3^ Department of Clinical Pharmacology CHU Nice and Université Côte d'Azur Nice France; ^4^ INSERM U1107 Migraine and Trigeminal Pain Auvergne University Clermont‐Ferrand France

**Keywords:** Base Nationale de PharmacoVigilance, cluster headache, COVID‐19 vaccines, disproportionality analysis, pharmacovigilance databases, VigiBase

## Abstract

**Objectives/Background:**

The main objective of our study was to assess the potential association between coronavirus disease 2019 (COVID‐19) vaccination and cluster headache (CH) using a pharmacovigilance approach. A secondary objective was to determine whether COVID‐19 vaccines predominantly triggered onset or worsening of the disease. Recent pharmacovigilance studies and case reports have suggested an association between COVID‐19 vaccines and cluster headache. In order to evaluate whether COVID‐19 vaccines could possibly elicit the onset of cluster headache or be responsible for worsening of the disease, we conducted a retrospective case–non‐case pharmacovigilance study using the international VigiBase pharmacovigilance database and analyzed the reported cases of cluster headache in the Base Nationale de Pharmacovigilance, French National Pharmacovigilance Database (BNPV).

**Methods:**

VigiBase was queried up to February 23, 2025, for all reports related to cluster headache and COVID‐19 vaccines. To evaluate the potential association, we performed a disproportionality analysis. This analysis relied on the calculation of the information component, for which a 95% confidence interval lower‐bound positivity was required for signal detection. The narratives of the reported cases of cluster headache in the BNPV were analyzed to determine whether COVID‐19 vaccination was responsible for the onset or worsening of the disease.

**Results:**

When focusing on the different COVID‐19 vaccines, Vaxzevria was found to be the strongest signal (IC_025_: 3.05). Lower signals were observed for the other COVID‐19 vaccines, including Comirnaty (IC025: 1.62), Spikevax (IC_025_: 1.24), and Jcovden (IC_025_: 0.05). The analysis of the 55 CH cases reported in the BNPV revealed a slight predominance of onset (24/55 cases classified as CH) over worsening of the disease (17/55 cases classified as CH).

**Conclusions:**

Our study supports the possibility of a potential association between COVID‐19 vaccines and cluster headache through an updated pharmacovigilance analysis. The signal appears to be more pronounced with the Vaxzevria vaccine.

## Plain Language Summary

Some recent reports suggest there might be a link between cluster headache and the coronavirus disease 2019 (COVID‐19) vaccination. To explore this, we carried out a global analysis using international and French drug safety monitoring databases and we found that cluster headache was reported more often than expected after COVID‐19 vaccination. Because cluster headache is considered as one of the most painful headache disorders, this possible link is important for healthcare professionals to be aware of and highlights the need for more research.

**Abbreviations**
ADR: Adverse drug reactions
BNPV: Base Nationale de Pharmacovigilance, French National Phramacovigilance Database
CGRP: Calcitonin gene‐related peptide
CCH: Chronic CH
CH: Cluster headache
COVID 2019: Coronavirus diesase 2019
ECH: Episodic CH
IC: Information component
PT: Preferred term
WHO: World Health Organization

## INTRODUCTION

Cluster headache (CH) is a rare primary headache disorder and its lifetime prevalence is estimated at 0.1% of the general population.^1^ Cluster headache, the most common of the trigeminal autonomic cephalalgias (TACs), is characterized by attacks of severe to very severe unilateral orbital, supraorbital, and/or temporal pain lasting from 15 to 180 min (when untreated), associated with ipsilateral autonomic symptoms and/or with restlessness or agitation. Attack frequency varies from one every other day to eight per day during cluster bouts and occur with pain‐free periods of at least 3 months in episodic CH (ECH) and without remission or with remissions lasting less than 3 months in chronic CH (CCH). Although a number of triggers have been described for attacks during an active phase, no specific triggers, apart from certain periods of the year related to the circannual periodicity of this primary headache, have been identified for the onset of ECH bouts or worsening of CCH. To our knowledge, such changes in the clinical expressivity of CH have not been reported as an adverse drug reaction (ADR) to any treatment.

In 2022, Brandt et al. reported seven Dutch patients with CH who had been attack‐free for an extended period of time (up to 7 years) and in whom a new cluster episode occurred within a few days after a COVID‐19 vaccination.^2^ Just after, Cheng et al. reported one Japanese patient and five Taiwanese patients with new CH bouts in close temporal relationships with COVID‐19 vaccination; two of them were de novo cases.^3^ A recent retrospective cohort study on patients with a history of CH revealed that, after COVID‐19 vaccination, patients experienced bouts that were more intense and lasted longer than those prior to vaccination.^4^

Beyond these data from tertiary centers, pharmacovigilance approaches have sought to clarify the link between COVID‐19 vaccination and CH. In a study focused on all neurological ADRs related to COVID‐19 vaccines collected internationally between December 15, 2020, and January 24, 2021, CH was found to be associated with COVID‐19 vaccines.^5^ Another international pharmacovigilance study focused on CH (reported on January 3, 2021) revealed a pharmacological signal associated with COVID‐19 vaccines in several hundred CH cases.^6^

We hypothesized that COVID‐19 vaccines are associated with a disproportionately higher number of spontaneous reports of CH compared to the background reporting of all other adverse drug reactions in the pharmacovigilance database. The aim of our study was to further assess through an updated international pharmacovigilance analysis the potential association between COVID‐19 vaccination and CH. The secondary objective was to determine whether COVID‐19 vaccines predominantly triggered onset or worsening of the disease.

## METHODS

### Study design

**FIGURE 1:**
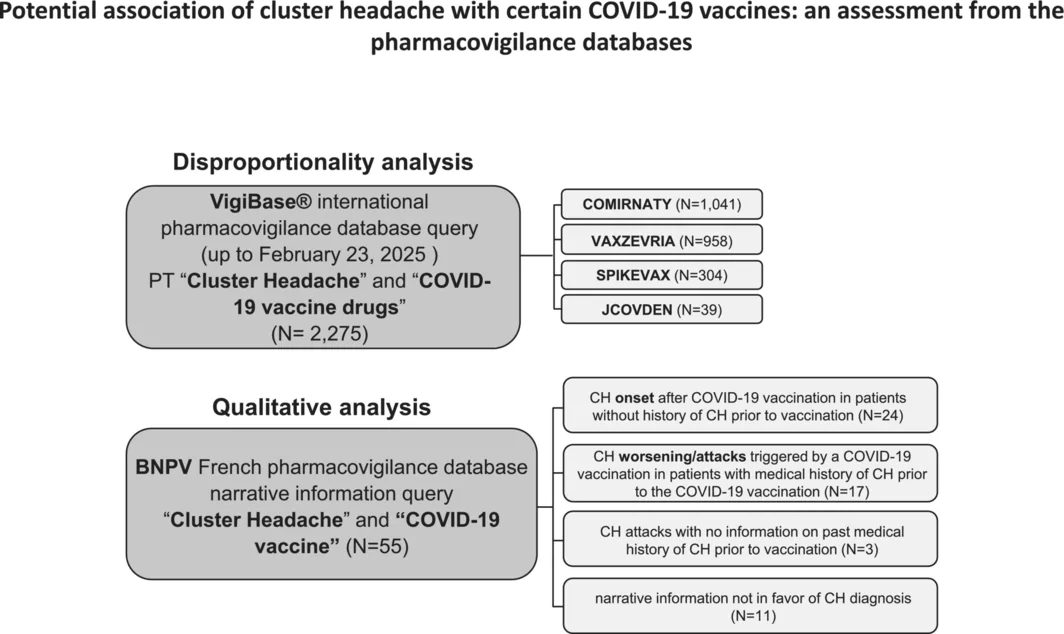
Flow diagram of the study design. To assess the possible association of COVID‐19 vaccination with CH, a disproportionality analysis (top) was performed using the VigiBase international pharmacovigilance database. A qualitative analysis (bottom) based on narrative information from 55 cases of CH following a COVID‐19 vaccination reported in the BNPV was conducted to determine if COVID‐19 vaccination could possibly trigger the onset or worsening of the disease. BNPV, Base Nationale de Pharmacovigilance, French National Pharmacovigilance Database; CH, cluster headache; COVID‐19, coronavirus disease 2019.

We carried out a retrospective case–non‐case study to analyze pharmacovigilance data concerning the association between CH and COVID‐19 vaccination collected internationally. Next, we performed a qualitative analysis of pharmacovigilance data collected in France to assess whether COVID‐19 vaccination could possibly trigger the onset or worsening of the disease. A flow diagram of the study is depicted in Figure 1.

### Quantitative analysis of CH associated with COVID‐19 vaccination

#### Data source

Quantitative analysis of CH associated with COVID‐19 vaccination was performed using VigiBase, the world's largest pharmacovigilance database set up by the World Health Organization (WHO).^7^ The data concerned were those collected between November 1, 1967 (first report in VigiBase), and February 23, 2025. Ethical approval was not required for the study involving humans in accordance with the local legislation and institutional requirements. Written informed consent to participate in this study was not required from the participants or the participants' legal guardians/next of kin in accordance with the national legislation and the institutional requirements. Notifications in the pharmacovigilance databases are anonymized at the time of local recording, with anonymity of both the patient and the notifier.

#### Queries

VigiBase was queried for all reports containing the preferred term (PT) “cluster headache” and concerning COVID‐19 vaccines. According to the Medical Dictionary for Regulatory Activities (version 27.1),^8^ a PT is defined as the distinct descriptor for a single medical concept. Deduplication of reports was performed automatically by VigiLyze. Only reports with more than 10 cases were retained for analysis. We opted for a higher threshold than the commonly used values of 3 or 5 because COVID‐19 vaccines generated a disproportionately high number of reports compared to other drugs.^9^, ^10^ This created a significant risk of background noise and false positives; our objective was therefore to maximize the specificity of our results.

For clarity, we have grouped under main brand names their different generic names of the COVID‐19 vaccines found in the database, as follows: Comirnaty (Tozinameran, Raxtozinameran, Bretovaneram) and Spikevax (Moderna, Andusomeran, Elasomeran, Davesomeran), which correspond to messenger RNA vaccines; Vaxzevria (ChAdOX1 nCOV19, NRVVAd) and Jcovden (JNJ, NRV Ad26), which are viral vector vaccines; and NOVAVAX (Nvx Cov2373), a protein subunit vaccine.

#### Disproportionality analysis

To determine whether CH was more frequently reported following COVID‐19 vaccination compared to all other combinations of ADRs and active ingredients, we performed a disproportionality^11^ analysis in VigiBase. This analysis helps to identify a potential pharmacological signal by comparing the observed number of chosen ADR occurring with a specific active ingredient with the expected number of ADR within the population based on the overall reporting of those ADRs. By doing so, we determined whether certain outcomes occurred more frequently than would be expected by chance. This disproportionality analysis was conducted using the information component (IC). This measure derives from a Bayesian confidence propagation neural network and is validated by the Uppsala Monitoring Center (UMC).^12^ The IC^8^ is a measure of the disproportionality between the observed and the expected reporting of a drug‐ADR pair. A positive IC value indicates that a particular drug‐ADR pair is reported more often than expected, based on all the reports in the database. Conversely, a negative IC value indicates that the drug‐ADR pair is reported less frequently than expected. IC_025_ and IC_975_ correspond to the 2.5 and 97.5th percentiles, respectively, of the posterior distribution of the IC. A positive value of IC_025_ is used as the threshold for identifying potential safety signals. The higher the value, the more the combination stands out from the background and the more statistically relevant the pharmacovigilance signal. Data were exported from VigiBase to Microsoft Excel (version 16.99.2; Microsoft, Redmond, WA, USA).

### Qualitative analysis of CH associated with COVID‐19 vaccination

Access to narrative information of cases reported in the French National Pharmacovigilance database (BNPV) allowed us to classify them into the following four categories: (1) “CH onset occurring after a COVID‐19 vaccination in a patient without history of CH prior to vaccination”; (2) “CH worsening/attacks triggered by a COVID‐19 vaccination in a patient with a medical history of CH prior to the COVID‐19 vaccination”; (3) “CH attacks with no information on whether the patient presented or not a past medical history of CH prior to vaccination”; and (4) “narrative information not in favor of a CH diagnosis.” The narrative descriptions were reviewed by two neurologists to determine whether they met the International Classification of Headache Disorders, 3rd edition, diagnostic criteria of CH^13^ and to which category they should be assigned. Inter‐rater reliability was assessed using Cohen's kappa. Analyses were performed using Python version 3.9.21 (Python Software Foundation, Wilmington, DE, USA) with the scikit‐learn library (version 1.5.2). Disagreement between raters was resolved by discussion to reach consensus. Deduplication of reports was performed by visual inspection. In addition, based on information recorded in the BNPV at the time of notification, data were collected on the clinical course of patients (recovered, recovery in progress, not recovered, or information not available), past medical history, and reported concomitant treatments when available. Skewed continuous variables are reported as medians with interquartile ranges and categorical variables as counts and percentages.

### Availability of data and materials

Two large pharmacovigilance databases were used in this study (Figure 1). The first is VigiBase, which includes part of the French cases but without narratives. The second is the BNPV, which includes narratives of the reported French cases needed for qualitative analysis. The data from VigiBase that support the findings of this study are available from the UMC. Access to the BNPV was granted via the Regional Pharmacovigilance Center in Lille and the Pharmacovigilance Center of Nice. The study results and conclusions expressed in this article are those of the authors and not necessarily those of the Uppsala Monitoring Center, the National Security Agency of Medicines and Health Products, the European Medicines Agency, the WHO, or one of their committees or working parties.

## RESULTS

### Quantitative analysis

#### Association of CH with COVID‐19 vaccines

A pharmacological signal was identified through the disproportionality analysis for COVID‐19 vaccines (IC025: 1.95) with 2275 reports, whereas 564 were expected (Table 1). When the analysis focused on the different COVID‐19 vaccines, a signal was observed for COVID‐19 vaccine Vaxzevria (IC_025_: 3.05). Lower signals were found for the other COVID‐19 vaccines, such as Comirnaty (IC_025_: 1.62), Spikevax (IC_025_: 1.24), and Jcovden (IC02_5_: 0.05) (Table 2).

**TABLE 1:** Disproportionality analysis of CH and COVID‐19 vaccines drugs in VigiBase.

Active ingredient	Reaction (PT)	*N*_observed_	*N*_expected_	IC_50_	IC_025_	IC_975_
**Covid‐19 vaccine**	**CH**	**2275**	**564**	**2.01**	**1.95**	**2.08**

*Note*: Only reports with ≥10 cases were analyzed. Positive IC_025_ values are indicated in bold.

Abbreviations: CH, cluster headache; COVID‐19, coronavirus disease 2019; PT, preferred term.

**TABLE 2:** Disproportionality analysis of CH and COVID‐19 vaccine drugs in VigiBase.

Drugs	Reaction (PT)	*N*_observed_	*N*_expected_	IC_50_	IC_025_	IC_975_
Vaxzevria	CH	958	106	**3.20**	**3.05**	**3.26**
Comirnaty	CH	1041	332	**1.71**	**1.62**	**1.89**
Spikevax	CH	304	111	**1.41**	**1.24**	**1.65**
Jcovden	CH	39	26	**0.57**	**0.05**	**0.98**

*Note*: Only reports with ≥10 cases were analyzed. Positive IC_025_ values are indicated in bold.

Abbreviations: CH, cluster headache; COVID‐19, coronavirus disease 2019; PT, preferred term.

### Qualitative analysis

With the aim of determining whether COVID‐19 vaccination triggers the onset or worsening of CH, we performed a qualitative analysis by examining the BNPV case narratives, an approach not feasible with Vigibase. A total of 55 cases of CH following a COVID‐19 vaccination were reported in the database, following removal of one duplicate case. Patients' characteristics are indicated in Table 3.

**TABLE 3:** Characteristics of the reported cases in the BNPV of CH occurring after COVID‐19 vaccination.

Characteristics	*N* = 55
Seriousness: *N* (%)	
Non‐serious	28 (51)
Serious	27 (49)
Vaccine: *N* (%)	
Comirnaty	37 (67)
Jcovden	2 (4)
Spikevax	11 (20)
Vaxzevria	5 (9)
Dose number: *N* (%)	
D1	31 (56)
D2	13 (24)
R1	11 (20)
Delay after vaccination in days: Median (min, max)	3 (0, 365) (*IQR*: 1–15)
Medical confirmation: *N* (%)	
Yes	12 (22)
No	10 (18)
Not reported	33 (60)
Sex: *N* (%)	
F	28 (51)
M	27 (49)
Median age in years: (min, max)	43 (21, 78), (*IQR*: 33–58)
Past medical history of CH: *N* (%)	
Yes	19 (35)
NO	27 (49)
Not reported	9 (16)
Other neurological past medical history: *N* (%)	
Yes	10 (18)
NO	21 (38)
Not reported	24 (44)
Other past medical history (other than neurological): *N* (%)	
Yes	22 (40)
NO	9 (16)
Not informed	24 (44)
Concomitant medications reported: *N* (%)	
Yes	12 (22)
NO	5 (9)
Not informed	38 (69)
Clinical evolution: *N* (%)	
Recovered; recovery in progress	9 (16); 10 (18)
Not recovered	31 (56)
Unknown	5 (9)
Reintroduction of a COVID‐19 vaccine: *N* (%)	
Yes with occurrence of CH	5 (9)
Yes without occurrence of CH	0
No	0
Not reported	50 (91)

*Note*: “Seriousness” is defined according to the following criteria: death, life‐threatening, caused or prolonged hospitalization, disabling/incapacitating, other medically important conditions. Events that did not meet any of these criteria are classified as “non‐serious.” Medical confirmation, reported as confirmed by a specialist.

Abbreviations: CH, cluster headache; COVID‐19; coronavirus disease 2019; D1 and D2, primary vaccination series; IQR, interquartile range; *n*, number; R1, booster doses.

Analysis of the narrative reports by two independent experts allowed cases to be classified into four categories, as shown in Figure 1. Agreement between raters across the four categories was substantial, as assessed using Cohen's kappa (κ = 0.65; 95% confidence interval [0.46, 0.80]).

The category distribution revealed a slight tendency toward the onset of the disease rather than worsening of the disease. Indeed, 24 of 55 cases (44%) corresponded to “CH onset appearing after a COVID‐19 vaccination in a patient without history of CH prior to vaccination”; 17 cases (31%) corresponded to “CH worsening/attacks triggered by a COVID‐19 vaccination in a patient with a medical history of CH prior to the COVID‐19 vaccination”; and three cases (5%) corresponded to “CH attacks with no information on whether the patient presented or not a past medical history of CH before vaccination.” Further, 11 cases (20%) could not be classified as the narrative information was not in favor of CH diagnosis.

Finally, we determined the distribution of the different vaccines across the four categories: “Cluster headache onset appearing after a COVID‐19 vaccination in a patient without history of CH prior to vaccination”: two with Vaxzevria, 16 with Comirnaty, six with Spikevax, 0 with Jcovden; “CH worsening/attacks triggered, by a COVID‐19 vaccination in patients presenting a medical history of CH prior to the COVID‐19 vaccination”: three with Vaxzevria, 12 with Comirnaty, one with Spikevax, one with Jcovden; “CH attacks with no information on whether the patient presented or not a past medical history of CH before vaccination”: 0 with Vaxzevria, two with Comirnaty, 0 with Spikevax, one with Jcovden; and “narrative information not in favor of CH”: 0 with Vaxzevria, seven with Comirnaty, four with Spikevax, 0 with Jcovden.

## DISCUSSION

By updating previous pharmacovigilance analyses, our findings are consistent with an association between COVID‐19 vaccines and CH, with the identification of a signal through the disproportionality analysis. When comparing the different COVID‐19 vaccines, the signal present for COVID‐19 vaccine appears to be more pronounced with Vaxzevria vaccine (IC_025_ = 3.05). In contrast, substantially lower signals were identified for the other vaccines, including Comirnaty (IC_025_ = 1.62), Spikevax (IC_025_ = 1.24), and Jcovden (IC_025_ = 0.05). Although the IC_025_ value for the Jcovden vaccine remains statistically positive, its magnitude is extremely close to the null value of zero, suggesting a very weak and borderline signal that is far less compelling than the strong signal observed for Vaxzevria.

Our results are consistent with the idea that COVID‐19 vaccines could possibly trigger CH attacks (onset or worsening of the disease). It does not seem that either mRNA‐based vaccines or viral vector vaccines prevail because signals were observed for both types.

To confirm these findings, and to quantify the incidence of CH triggered by COVID‐19 vaccines, prospective studies and follow‐up epidemiological studies should be conducted. As CH is known to present an annual rhythmicity, it would be interesting to investigate through a prospective study if the time of the year has an impact on the association between COVID‐19 and CH. To our knowledge, no treatments are known to date to trigger CH similarly to COVID‐19 vaccine. It should be noted that the intake of alcohol can trigger attacks during the active phase of some patients.^14^ Glyceryl trinitrate has been demonstrated in non‐placebo–controlled studies to induce CH during the active phase in episodic patients and in patients with chronic CH but, surprisingly, not in the remission‐phase of patients with episodic CH.^15^, ^16^ Similar results have been found with calcitonin gene‐related peptide (CGRP) because it has been shown to provoke CH attacks in active phase of episodic and chronic CH but not during the remission phase in episodic CH.^17^ When examining the BNPV cases, COVID‐19 vaccines unlike CGRP and glyceryl trinitrate appear to trigger a worsening in CH patients in remission but also trigger new onset of CH; however, these findings are limited by the small number of reported cases and the lack of detailed clinical data describing them, which makes it difficult to extend these results to a larger population.

We know that CH pathogenesis involves complex interactions between the trigeminovascular pathway, trigeminal autonomic reflex, hypothalamus, and neuropeptides such as CGRP and pituitary adenylate cyclase‐activating polypeptide 38. The mechanism of how COVID‐19 vaccine can possibly trigger CH attacks is unknown.^18^ The effectiveness of corticosteroids on CH bouts highlights the possible role of inflammation in CH pathophysiology. This hypothesis is supported by the reduction in CGRP plasma concentration and frequency of the attacks following corticoid administration.^19^ Considering this, COVID‐19 vaccines could possibly trigger episodes of CH through an inflammatory process. In this context, it is hypothesized that patients with CH present a fluctuating proinflammatory state in the trigeminovascular system and that the immune response elicited by vaccination could activate this system. Release of prostaglandins and bradykinins by immune cells could possibly lead to CGRP release and induce the inflammatory state responsible for CH attacks.

When headaches occur following a COVID‐19 vaccination in patients with a history of migraine, these headaches tend to be more intense in severity, longer in duration,^20^, ^21^ and more frequently resemble “a migraine‐like type headache.” This contrasts with our analysis of the BNPV, in which disease onset appeared to be more frequently reported than disease worsening; however, our study did not allow us to evaluate the duration nor the severity of the headache. Our findings neither undermine the benefit/risk ratio of the vaccines nor the importance of COVID‐19 vaccination. In addition to the progressive decline in reporting over time, particularly for well‐known or non‐serious events (a phenomenon often referred to as *declaration fatigue*), the number of reported CH attacks is expected to decrease. This trend is anticipated to be accentuated by recent changes in COVID‐19 vaccination practices in line with the evolution of government vaccination strategies (e.g., CDC 2025–2026 COVID‐19 vaccination guidance in the United States),^22^ in which COVID‐19 immunization is no longer systematically recommended for the general young adult population but is primarily targeted toward older individuals (aged ≥65 years) and those at high risk. Consequently, vaccine exposure among younger populations has progressively declined, which may contribute to a concomitant reduction in vaccination‐associated adverse events reported in this age group, including CH attacks. To better understand the possible association between CH and COVID‐19 vaccines, it is crucial to encourage reporting of CH attacks following a COVID‐19 vaccination.

Our findings provide valuable insights into a potential association between CH and certain COVID‐19 vaccines; however, they should be interpreted in light of certain limitations. The presence of a signal does not establish causality, and the possibility of an association may be coincidental. Patients might have experienced the adverse event regardless of vaccination, and the heightened scrutiny of vaccine safety may have led to vaccines being wrongly implicated. The presence of a signal with CH and COVID‐19 vaccine could possibly be explained by a Weber effect,^23^ driven by increased reporting of ADR due to heightened public awareness and media attention. It should be noted that a case‐by‐case analysis is not possible on the basis of VigiBase. This limitation justified our decision to cross‐check with the French pharmacovigilance database, in which narrative descriptions allowed verification of the accuracy of the CH diagnosis. Indeed, as a result, 20% of reported cases could not be classified because the narrative information was not in favor of a CH diagnosis, an important proportion, which clearly illustrates the limitations of pharmacovigilance database approaches when narrative clinical details are lacking. Whereas the BNPV provides access to supplementary information through narrative case descriptions, missing data were frequent, particularly regarding concomitant treatments, past medical history, and patients' clinical evolution^24^—and in some cases, clinical description of the cases was scant. In addition, despite the availability of narrative information in the BNPV, the possibility of misclassification, misdiagnosis, or underreporting of prior CH history cannot be excluded in our qualitative analysis, the latter of which may have resulted in an overestimation of the proportion of cases classified as “onset.” Conversely, underreporting of CH is also likely because CH remains underdiagnosed^25^ due to its rarity and the limited awareness within the medical community. Moreover, underreporting is an inherent limitation of studies based on spontaneous reporting. Finally, the small number of reported cases and lack of detailed clinical data further limit the possibility of extrapolating our results to a broader population.

## CONCLUSIONS

Through an updated international pharmacovigilance analysis, our study supports the possibility of an association between COVID‐19 vaccination and CH. The signal present for COVID‐19 vaccine appears to be more pronounced with the Vaxzevria vaccine. Nonetheless, the disproportionality signals observed in this study require confirmation through other study designs. In addition, analysis of reported cases of CH attacks in the French pharmacovigilance database indicates a potential association with disease onset rather than a worsening of preexisting CH, a finding that, despite being constrained by the completeness of available case data, highlights an important avenue for further investigation.

## AUTHOR CONTRIBUTIONS

**Elise K. Van Obberghen:** Conceptualization; writing – original draft; methodology; validation; writing – review and editing; investigation. **Laura Parigny:** Data curation; writing – review and editing. **Nouha Ben‐Othman:** Writing – review and editing; data curation. **Elliot Ewig:** Writing – review and editing; data curation. **Milou‐Daniel Drici:** Writing – review and editing; conceptualization; methodology; supervision. **Alexandre O. Gérard:** Writing – review and editing; data curation; methodology. **Sophie Gautier:** Supervision; conceptualization; writing – review and editing. **Michel Lanteri‐Minet:** Conceptualization; methodology; validation; supervision; writing – review and editing; writing – original draft.

## FUNDING INFORMATION

The authors declare that no financial support was received for the research, authorship, and/or publication of this article.

## CONFLICT OF INTEREST STATEMENT

**Elise K. Van Obberghen**, **Laura Parigny**, **Nouha Ben‐Othman**, **Elliot Ewig**, **Milou‐Daniel Drici**, **Alexandre O. Gérard**, **Sophie Gautier**, and **Michel Lanteri‐Minet** declare no conflicts of interest.
